# T2D and Depression Risk Gene Proteasome Modulator 9 is Linked to Insomnia

**DOI:** 10.1038/srep12032

**Published:** 2015-07-13

**Authors:** Han Hao, Michael J. Haas, Rongling Wu, Claudia Gragnoli

**Affiliations:** 1 Department of Statistics, Penn State University, State College, PA, USA; 2 Division of Endocrinology, Diabetes, and Metabolism, Department of Medicine, University of Florida College of Medicine, Jacksonville, FL; 3 Department of Public Health Sciences, Penn State College of Medicine, Hershey, PA, USA; 4 Center for Biotechnology and Department of Biology, Temple University’s College of Science & Technology, Philadelphia, PA, USA; 5 Molecular Biology Laboratory, Bios Biotech Multi-Diagnostic Health Center, Rome, Italy

## Abstract

Insomnia increases type-2 diabetes (T2D) risk. The 12q24 locus is linked to T2D, depression, bipolar disorder and anxiety. At the 12q24 locus, the Proteasome-Modulator 9 (*PSMD9)* single nucleotide polymorphisms (SNPs) rs74421874 [*intervening sequence* (*IVS*) *3+nt460-G>A*], rs3825172 (*IVS3+nt437-C>T*) and rs14259 (*E197G-A>G*) are linked to: T2D, depression, anxiety, maturity-onset-diabetes-of the young 3/MODY3, obesity, waist circumference, hypertension, hypercholesterolemia, T2D-macrovascular disease, T2D-microvascular disease, T2D-neuropathy, T2D-carpal-tunnel syndrome, T2D-nephropathy, T2D-retinopathy and non-diabetic retinopathy. *PSMD9 SNP* rs1043307/rs14259 (*E197G-A>G*) plays a role in anti-depressant therapy response, depression and schizophrenia. We aimed at determining *PSMD9* rs74421874/rs3825172/rs14259 SNPs potential linkage to primary insomnia and sleep hours in T2D families. We recruited 200 Italian T2D families phenotyping them for primary insomnia and sleep hours per night. *PSMD9*-T2D-risk SNPs rs74421874/rs3825172 and rs1043307/rs14259 were tested for linkage with insomnia and sleep hours. Non-parametric-linkage analysis, linkage-disequilibrium-model analysis, single-SNP analysis, cluster-based-parametric analysis, quantitative-trait and variant-component analysis were performed using Merlin software. To validate data, 1000 replicates were executed for the significant non-parametric data. *PSMD9* rs74421874 (*IVS3+nt460-G>A*), rs3825172 (*IVS3+nt437-C>T*) and rs1043307/rs14259 (*E197G-A>G*) SNPs are linked to insomnia in our Italian families.

Insomnia is associated with increased risk for type-2 diabetes (T2D)^1^. It is known that deficiency of sleep/insufficient sleep schedules and/or insomnia with short-sleep duration contribute to glucose homeostasis impairment. Sleep deficiencies associated with metabolic dysregulation leads to obesity and T2D^2^. In a study it was shown that 48.2% of T2D patients had insomnia^3^. Furthermore, glucose metabolism can be negatively impaired by sleep issues such as obstructive sleep apnea (OSA), which causes insulin resistance and alters glucose metabolism. Sleep duration and metabolic syndrome are correlated; OSA and T2D share several impaired metabolic characteristics. Sleep deprivation, sleep disordered breathing-OSA and insomnia are strongly associated with T2D. OSA treatment increases insulin sensitivity and improves glucose metabolism^4^. Chronic sleep restriction in rats induces metabolic dysfunction, increased plasma glucose, fasting insulin, lipids, insulin resistance and oxidative stress and decreases glucose tolerance; these parameters improve by treatment with piromelatine, a novel melatonin agonist, used for insomnia. Piromelatine given to high-fat/high-sucrose-fed rats has shown positive benefits in sleep maintenance, anxiety, depression, weight gain inhibition, and increase in insulin sensitivity, antioxidative potency, improvement of glucose and lipids levels, and of glucose tolerance^5^. T2D is associated with an increase in the prevalence of psychiatric disorders^6^,^7^,^8^,^9^,^10^. These above-mentioned factors lead to the hypothesis that a genetic predisposition to insomnia may confer increase risk for T2D.

Within the 12q24 locus^11^ lies the gene of Proteasome-Modulator 9 (*PSMD9)*, contributing to T2D through rare variants [rs149556654 SNP (*N166S A>G*) and *S143G A>G* variant]^12^. *PSMD9* is significantly linked via the common single nucleotide polymorphisms (SNPs) rs74421874 (*IVS3+nt460 G>A*), rs3825172 (*IVS3+nt437 C>T*) and rs14259 (*E197G A>G*) to T2D^13^; MODY3^14^; T2D-neuropathy, T2D-nephropathy, T2D-retinopathy and non-diabetic retinopathy; T2D-microvascular^15^,^16^,^17^,^18^ and T2D-macrovascular complications (stroke, coronary artery disease, myocardial infarction and vasculopathy)^19^; carpal tunnel syndrome^20^; hypercholesterolemia^21^; overweight condition and waist circumference^22^; hypertension^23^; depression^24^, and anxiety^25^. Of note, the 12q24 locus is linked to T2D^11^; macrovascular^26^,^27^, and microvascular disease^28^; dyslipidemia^29^; hypertension; obesity^30^, body mass index and C-reactive protein jointly^31^; bipolar disorder^32^, depression^33^, and anxiety^34^. A depression and anxiety risk gene, given possible common pathogenesis among depression, anxiety and insomnia, can also be a candidate gene for insomnia. Furthermore, *PSMD9* SNP rs1043307/rs14259 (*E197G A>G*) is implicated in anti-depressant therapy response^35^, *PSMD9* SNPs rs74421874 (*IVS3+nt460 G>A*), rs3825172 (*IVS3+nt437 C>T*) and rs14259 (*E197G A>G*) are linked to depression^24^ and anxiety^25^, and *PSMD9* is strongly implicated in depression^36^ and schizophrenia^37^; thus, as all these disorders may share a common pathogenic inflammatory pathway, *PSMD9* may well play a role in insomnia.

PSMD9 is ubiquitously expressed and highly concentrated in eukaryotic cells as part of the 26S proteasome complex, which degrades intracellular proteins into antigenic peptides in the immune response and antigen presentation by MHC class I cells (http://www.genecards.org/cgi-bin/carddisp.pl?gene=PSMD9&search=psmd9). Therefore, PSMD9 may have a pathogenic role in inflammation and autoimmunity^38^. The 26S proteasome is also a factor regulating ligand-dependent retinoid-target genes transcription, and PSMD9 PDZ interaction domain has a role in mRNA processing and editing, transcriptional regulation, hormone and receptor activity and protein translation^39^. Additionally, novel PSMD9-interacting partners have been recently identified: hnRNPA1 (an RNA binding protein), S14 (a ribosomal protein), CSH1 (a growth hormone), E12 (a transcription factor) and IL6 receptor^39^. This has added to our understanding of the PSMD9 role in regulation of transcription, processing and editing of mRNA, protein translation, hormone and receptor activity^39^. Another study demonstrated that PSMD9 inhibits basal and tumor necrosis factor α (TNF-α)-mediated NF-kB activation via inhibition of nuclear factor kB α (IkBα) proteasome degradation^40^. Thus, *PSMD9* variants impairing PSMD9 protein sequence and/or protein dosage may alter downstream effects of its functions as well as interaction with key receptors such as the IL6 receptor. As PSMD9 is part of a complex network of transcription and co-activators, reduced or increased *PSMD9* gene may lead to different phenotypes, or to an underlying pathological factor such as inflammation contributing to the various phenotypes, and commonly shared by them. Its proven interaction with the IL6 receptor may potentially explain its pleiotropic role in multiple disorders, whose pathogenesis is mediated by inflammation. However, other possible explanations for a shared pathogenesis among neuro-psychiatric disorders exist. For example, adult neurogenesis might be a more plausible pathogenic mechanism for neuro-psychiatric disorders. The proteasome-mediated proteolysis plays a recognized role in neuronal development, synaptic plasticity, and protein quality control. A proteomic study has reported the positive finding of PSMD9 at both the level of neuronal cytosol as well as synapsis^41^. In addition, *PSMD9* gene role in the ubiquitin-proteasome system and its proven protein interaction with dysbindin and dysbindin-related role in neuroplasticity is intriguing^42^. Also PSMD9 role in cell cycle and mitosis opens up to other potential avenues for its patho-mechanicistic role^43^.

In light of these data, our object in the present study was to test the *PSMD9-*depression-risk SNPs rs74421874 (*IVS3+nt460 G>A*), rs3825172 (*IVS3+nt437 C>T*) and rs14259 (*E197G A>G*) for linkage with insomnia and sleep hours in the 200 Italian T2D families.

## Material and Methods

### Ethical Statement

The subjects were all recruited from central Italy following the Helsinki-declaration guidelines. Subjects gave written informed consent. The Penn State Institutional Review Board approved the study.

### Families

The Italian T2D families with affected sib-pairs were recruited via standardized interview from Rome. They are genetically homogeneous, and all have been Italian for at least three generations; T2D families have a T2D-family history. We excluded families with identical twins and siblings with uncertain identical paternity. The Italian family dataset has provided useful genetic information in T2D and associated phenotypes^12^,^44^,^45^,^46^,^47^,^48^,^49^,^50^,^51^,^52^,^53^,^54^,^55^,^56^,^57^,^58^,^59^,^60^,^61^.

The Italian T2D families with mainly affected sib-pairs were recruited via standardized interview. The 200 Italian families are mainly constituted by 360 ungenotyped parents of 180 affected sib-pairs (163 affected sib-pairs, 15 affected triplet sib-pairs 1 affected quaternal sib-pairs, 1 affected penta sib-pairs) for a total of 380 affected siblings, plus 20 families inclusive of 101 genotyped family members with an average family size of 5.05. The total of genotyped family members is 481, including 245 female subjects and 236 male subjects. The median age was 63. T2D was diagnosed based on the fasting criteria of blood glucose ≥126 mg/dl on at least two occasions, or a random glucose of ≥200 mg/dl associated with symptoms of diabetes, exclusion of type 1 diabetes, and a positive family history for non-insulin-dependent diabetes/T2D. T2D was present in 94.10% of the families’ members. Major depression and generalized anxiety disorder were diagnosed based on the subject meeting the DSM-IV criteria during his/her lifetime. Generalized anxiety disorder was present in 52.50% of families and major depression in 29.90% of families.

We characterized the Italian families for the presence or absence of insomnia based on the DSM-IV diagnosis criteria for primary insomnia; the subject needed to have had in the past year trouble falling or staying asleep or not feeling rested waking up for at least one month; this had to significantly interfere with the routine activities; also any medical condition (e.g., hyperthyroidism or drug abuse), which may cause this similar status, was excluded. If the subjects were negative or positive for the DSM-IV diagnostic criteria for generalized anxiety disorder or major depression that was annotated. Other mental disorders were excluded. Further, an average sleep time in the past year of less than four hours per night was considered insomnia. Of the very few patients with insomnia on sleep medication, we considered only the average sleep hours before starting the medication, independently on when the medication was started. Primary insomnia was present in 42.20% of families.

The phenotype of insomnia is described as unknown if data are lacking. We could not collected insomnia data for all of the members in our 200 Italian families.

### Sequencing

Using polymerase chain reaction (PCR) and with specific primers, we amplified in all family members the *IVS3*-*PSMD9* region containing the SNPs rs74421874 (*IVS3+nt460 G>A*), rs3825172 (*IVS3+nt437 C>T*) and the exon 5-coding region, containing the rs1043307/rs14259 (*E197G A>G*). We directly sequenced the PCR products, post-purification via EXOSAP-IT, on an automated ABI 3730 Sequencer.

### Statistical Analysis

In the 200 Italians families we tested the *PSMD9* SNPs rs74421874 (*IVS3+nt460 G>A*), rs3825172 (*IVS3+nt437 C>T*) and rs1043307/rs14259 (*E197G A>G*) for linkage with primary insomnia and average sleep hours per night. Both non-parametric and parametric-linkage analysis for the qualitative phenotype were performed for the three SNPs, using Merlin software^62^. Allele frequencies were calculated from the real data^62^. If Merlin demonstrated that a family is not informative given the clinical phenotype and genetic data available, while the genetic data are used to compute the overall allelic frequency, the family was excluded from the linkage analysis.

We previously reported that the *PSMD9* SNPs rs74421874 (*IVS3+nt460 G>A*), rs3825172 (*IVS3+nt437 C>T*) and rs1043307/rs14259 (*E197G A>G*) are in strong linkage disequilibrium (LD)^12^. The linkage is inflated by linkage disequilibrium if SNPs are analyzed jointly, and uninfluenced if SNPs are analyzed independently. To eliminate the LD influence of the variants, we performed LD-modeling non-parametric linkage analysis, which takes into consideration the haplotype clusters. Furthermore, we tested each single SNP independently under the non-parametric model to eliminate the potentially confounding LD-based influence on the results.

In order to rule out the effect of depression or anxiety on insomnia, we also performed a likelihood ratio test of each SNP association with insomnia considering anxiety or depression as a covariate, by using a mixed effects logistic regression model taking into account the families as a random effect factor in the model. For the Chi-square statistics, a corresponding p-value less than 0.05 means that the SNP tested has a significant effect on insomnia in the presence of the tested covariate and families as a random effect. The p-value is calculated by performing a likelihood ratio test, where the test statistic is the Chi-square statistic, which has a degree of freedom “DF” depending on the covariate and random effects.

The following parameters were used for the parametric-linkage analysis based on the SNPs cluster, thus eliminating the LD inflation of the linkage signal, as it considers the SNPs all in the same locus: cluster haplotype disease-allele frequency 0.25; dominant model with penetrance for homozygous non-risk allele 0.13 (equal to the prevalence of insomnia in the Italian population), for heterozygous risk allele 1.00, for homozygous risk allele 1.00; recessive model with penetrance for homozygous non-risk allele 0.13, for heterozygous risk allele 0.13, for homozygous risk allele 1.00; additive model with penetrance for homozygous non-risk allele 0.13, for heterozygous risk allele 0.45, and for homozygous risk allele 0.90. Merlin computed the lod score from all the informative families for the insomnia phenotype (n = 28).

For each significant non-parametric test performed, we calculated how many times similar p-values were expected by chance in 1,000 replicates of simulations using the gene-dropping method in order to exclude the presence of any false positives in our results. This analysis replaces real data with simulated data, while maintaining the pedigree structure, allele frequencies, and recombination fraction. These datasets were generated under the null hypothesis of no linkage.

The quantitative-trait linkage analysis and variance-component analysis for the quantitative trait of average sleep hours per night was performed using Merlin^62^. Merlin computed the lod score from all the informative families for the average sleep hours (n = 60 for the quantitative-trait test and n = 118 for the variance component test).

All results are reported as LOD scores as calculated by Merlin.

## Results

Our analysis shows that the *PSMD9* SNPs rs74421874 (*IVS3+nt460 A>G*), rs3825172 (*IVS3+nt437 C>T*) and rs1043307/rs14259 (*E197G A>G*) are linked to primary insomnia via the non-parametric model (LOD = 3.16, p-value = 0.00006).

Additionally, we identified a positive non-parametric linkage of the above-mentioned *PSMD9* SNPs to primary insomnia by using the LD-based model analysis and by testing each single SNP independently. The simulation analyses of 1,000 replicates exclude false positives and establish the data validity. The result of the non-parametric linkage analysis for primary insomnia are reported in Table 1.

The Chi-square statistics reveals a significant association of *PSMD9* SNP rs74421874 *(IVS3* *+nt460G>A*) and SNP rs3825172 (*IVS3* *+nt437C>T*), independently, with insomnia using anxiety as covariate (p-value = 0.029), but not using depression as covariate (p-value = 0.10). The Chi-square statistics also shows a significant association of rs1043307/rs14259 (*E197G A>G*) with insomnia using anxiety as covariate (p-value=0.010) as well as using depression as covariate (p-value = 0.027, Table 2).

The PSMD9 SNPs cluster results in linkage with primary insomnia under the recessive inheritance model. The outcome of the parametric linkage tests are described in Table 3.

The variance component analysis shows a trend of the sleep hours-related trait towards linkage with a p-value = 0.06 and a 1,000 simulation-derived empirical p-value = 0.006; this means that there are six chances in 1,000 that the variance component data are due to random chance. The data outcome of the variance-component analysis is reported in Table 4.

The quantitative trait analysis testing sleep hours reports a linkage signal with corresponding p-value = 0.006 (Table 5).

## Discussion

Our study demonstrates that the *PSMD9* SNPs rs74421874 (*IVS3+nt460 G>A*), rs3825172 (*IVS3+nt437 C>T*) and rs1043307/rs14259 (*E197G A>G*) studied are linked to primary insomnia in the Italian T2D families; it shows a trend towards linkage to sleep hours, with a trait heritability estimated to be circa 11%, and gene-trait heritability circa 25%. The different types of non-parametric-linkage analyses performed determined that the linkage is present independently from the presence of LD among variants; the simulations excluded the possibility that the results are due to random chance.

The logistic regression model supports the role of the *PSMD9* coding variant rs14259 (*E197G A>G*) for a significant association with insomnia, considering anxiety or depression as a covariates. The two *PSMD9* intronic SNPs rs74421874 (*IVS3+nt460 G>A*) and rs3825172 (*IVS3+nt437 C>T*) are significantly associated with insomnia considering anxiety as covariate, but lose significance in the association test with insomnia considering depression as covariate. Thus, the *PSMD9* intronic variants rs74421874 (*IVS3+nt460 G>A*) and rs3825172 (*IVS3+nt437 C>T*) may provide vulnerability to the missense coding variant rs1043307/rs14259 *E197G*, which by itself may be the pathogenic variant.

In addition, we identified the recessive model as the most significant for the linkage, as our families’ structure contain sib-pairs, which are the most statistically powerful family dataset to identify recessive disorders.

Our study may have also captured the linkage signal due to the presence of T2D; however, this risk is always present if we test for linkage in a complex disorder which is associated with another common disorder. Nevertheless, the variance-component analysis of sleep hours, based on a quantitative trait independent from the T2D qualitative phenotype, shows at least a trend towards linkage of sleep hours.

As insomnia is a risk factor for T2D, it is reasonable to anticipate that a gene may be linked to both insomnia and T2D. Furthermore, *PSMD9* is a ubiquitously expressed gene, which is implicated in anti-depressant therapy response and depression^24^,^35^,^36^. The potential role of PSMD9 for the anti-depressant therapy effects and its implication in depression^36^ and schizophrenia^37^ are likely due to its function in the inflammatory process. Thus, PSMD9 mediating inflammation may also contribute to insomnia.

The IL6 receptor has been identified as a novel PSMD9-interacting partner which further supports PSMD9 role in the inflammatory response^39^. In addition, PSMD9 mediates IκBα degradation via PSMD9-PDZ domain-specific interaction and a hnRNPA1 C-terminus motif 40. Point mutations in the PDZ domain or deletion of amino acid residues from the hnRNPA1 C-terminal residues disrupt the interactions between the two proteins, thereby affecting NF-κB activity^40^. These findings corroborate the hypothesis of how a PSMD9 SNP may play a pathogenic inflammatory risk role and thus a pleiotropic effect on different phenotypes.

In summary, *PSMD9* is linked to primary insomnia in our T2D Italian families. Specifically, the *PSMD9* coding variant rs1043307/rs14259 *E197G* is significantly associated with insomnia taking into account anxiety or depression as covariate, while the *PSMD9* intronic SNPs rs74421874 (*IVS3+nt460 G>A*) and rs3825172 (*IVS3+nt437 C>T*) remain significantly associated with insomnia only when taking into account anxiety –and not depression– as covariate. These findings are new, and should be independently replicated in other populations. These data, if confirmed in other populations, imply that PSMD9-targeted therapies may prevent or treat primary insomnia, depression, and T2D, and/or to manage personalized therapy based on patient genotypes.

## Additional Information

**How to cite this article**: Hao, H. *et al.* T2D and Depression Risk Gene Proteasome Modulator 9 is Linked to Insomnia. *Sci. Rep.* **5**, 12032; doi: 10.1038/srep12032 (2015).

## Figures and Tables

**Table 1:** Non-Parametric and Linkage-Disequilibrium-Based-Model Analysis of Primary Insomnia of the 200 Italian Families by Merlin software.

**Insomnia**	**Prevalence**	**Families**	**Lod Score**	**P**	**Empirical P**
Non-Parametric-Joint SNPs	42.20%	28	3.16	0.00006	0.000999
Linkage-Disequilibrium-Model	42.20%	28	0.88	0.02	0.03
SNP *IVS* 3*+nt460G>A (MAF = A 30%)*	42.20%	28	1.28	0.01	0.027
SNP *IVS* 3*+nt437C>T (MAF = T 30%)*	42.20%	28	1.28	0.01	0.022
SNP *E197G A>G (MAF = G 30%)*	42.20%	28	1.03	0.02	0.031

MAF = minor allele frequency. Prevalence = phenotype prevalence among the family subjects studied; Families = families number analyzed; Lod score = derived from the linkage analysis by Merlin; P = p-value; Empirical P = p-value derived from 1,000 replicates by using the gene dropping method.

**Table 2:** Chi-Square Statistics of Primary Insomnia of the 200 Italian Families (Anxiety or Depression as Covariate).

**SNP**	**Covariates**	**Random Effect**	**Chi-Square Statistic**	**DF**	**P**
*IVS* 3*+nt460G>A*	Anxiety	Family	18.548	9	0.029
*IVS* 3*+nt460G>A*	Depression	Family	14.671	9	0.100
*IVS* 3*+nt437C>T*	Anxiety	Family	18.548	9	0.029
*IVS* 3*+nt437C>T*	Depression	Family	14.671	9	0.100
*E197G A>G*	Anxiety	Family	21.671	9	0.010
*E197G A>G*	Depression	Family	18.776	9	0.027

DF = degree of freedom; P = p-value.

**Table 3:** Parametric-Linkage Analysis of Primary Insomnia in the 200 Italian Families by Merlin software.

**Parametric Analysis**	**Prevalence**	**Families**	**Lod Score**	**P**
Dominant Model; SNPs Cluster	42.40%	28	−3.339	−
Recessive Model; SNPs Cluster	42.40%	28	1.173	0.020207
Additive Model; SNPs Cluster	42.40%	28	0.605	0.09527

Prevalence = phenotype prevalence among the family subjects studied; Families = families number analyzed; Lod score = derived from the cluster-based parametric-linkage analysis by Merlin; P = p-value.

**Table 4:** Variance-Component Linkage Analysis of Sleep Hours of the 200 Italian Families by Merlin software.

**Quantitative Trait**	**Families**	**Trait Heritability**	**Gene-Trait Heritability**	**Lod Score**	**P**	**Empirical P**
Sleep Hours	118	11.48%	25.92%	0.54	0.06	0.006

Families = families number analyzed; Lod score = derived from the quantitative-trait analysis by Merlin; P = p-value; Empirical P = p-value derived from 1,000 replicates by using the gene-dropping method; Trait Heritability = Heritability of the quantitative trait calculated; Gene-Trait Heritability = Heritability of the quantitative trait attributable to the *PSMD9* SNPs.

**Table 5:** Quantitative-Trait Linkage Analysis of Sleep Hours of the 200 Italian Families by Merlin software.

**Quantitative Trait**	**Families**	**Lod Score**	**P**
Sleep Hours	60	1.39	0.006

Families = families number analyzed; Lod score = derived from the quantitative-trait analysis by Merlin; P = p-value.
